# Mapping cross-reactive residues in the G-H loop of foot-and-mouth disease virus: insights for serotype-specific design

**DOI:** 10.3389/fmicb.2025.1631386

**Published:** 2025-07-23

**Authors:** Abdelaziz A. Yassin, Anna B. Ludi, Santina Grazioli, Emiliana Brocchi, Daniel Horton, Donald P. King, Amin S. Asfor

**Affiliations:** ^1^The Pirbright Institute, Pirbright, Woking, Surrey, United Kingdom; ^2^Department of Comparative Biomedical Sciences, School of Veterinary Medicine, University of Surrey, Guildford, United Kingdom; ^3^Department of Foot and Mouth Disease, Veterinary Serum and Vaccine Research Institute, Cairo, Egypt; ^4^Istituto Zooprofilattico Sperimentale della Lombardia e dell’Emilia Romagna (IZSLER), Brescia, Italy; ^5^Animal and Plant Health Agency, Weybridge, United Kingdom

**Keywords:** FMDV, overlapping, alanine scanning, G-H loop neutralising epitope, G-H loop peptides, inter-serotypes cross-reactivity, D9 and B2 Mab

## Abstract

**Introduction:**

Foot-and-mouth disease virus (FMDV) has a hypervariable G-H loop region within the VP1 capsid protein. This structure is associated with virus neutralisation and contains the virus attachment motif (RGD) which binds to the cellular integrin receptor facilitating virus entry for all seven FMDV serotypes.

**Methods:**

Six monoclonal antibodies (Mabs) were tested against 10 peptides representing the wild-type G-H loops of serotypes O, A, SAT1, and SAT2. D9 and B2 Mabs were raised against serotype O and tested against three more sets of peptides: (1) nine overlapping peptides with one amino acid difference, (2) alanine scanning peptide, both for O1K strain and (3) four mutated peptides one for A22 and three for SAT2 strains in the enzyme-linked immunosorbent assay format with correlation to virus neutralisation test.

**Results:**

The D9 Mab was bound to peptides corresponding to the G-H loops of serotype O, A, and SAT1 strains, but only neutralised serotype O and SAT1 strains in the virus neutralisation test. The B2 Mab is also bound to serotype O and SAT1 but only neutralised serotype O. Using a set of overlapping peptides, the binding region for the D9 Mab was confirmed as amino acid positions 144, 147 and 148. An additional critical amino acid residue at position 145R was identified using a set of alanine scanning peptides. The binding region for B2 Mab appears to be upstream of RGD as B2 showed lower binding to peptides lacking the first three amino acids of the GH-loop peptides. These critical amino acids were further confirmed by designing modified SAT2 and A peptides at these positions, which led to a significant improvement in the binding ability of D9 but not B2.

**Conclusion:**

These findings help to map cross-reactive epitopes within the G-H loop which may contribute to the inter-serotypic cross-reactivity observed in diagnostic serological assays giving insights that motivate the design of custom peptides that have improved serotype specificity.

## 1 Introduction

Foot-and-mouth disease virus (FMDV) is a member of *Aphthovirus* genus in the order *Picornavirales* within the family *Picornaviridae*. It is a non-enveloped virus with an icosahedral capsid and a positive sense single-stranded RNA genome of approximately 8,400 nucleotides (Forss et al., 1984). The virus is about 30 nm in diameter and contains 60 copies of each of the four proteins VP1 (1D), VP2 (1B), VP3 (1C), and VP4 (1A). VP1, VP2 and VP3 are located on the surface whilst VP4, a smaller protein, is on the inside of the capsid (Acharya et al., 1989). The VP1 proteins are located around the five-fold axes whilst the VP2 and VP3 proteins alternate around the three-fold axes of the capsid (Mahy, 2005). There are seven antigenically distinct serotypes: O, A (Vallée and Carré, 1922), C (Waldmann and Trautwein, 1926), Asia 1 (Dhanda et al., 1957) and Southern African Territories (SAT)1, SAT2 and SAT3 (Brooksby, 1958). VP1 is the most variable region of the genome with an average of 86% sequence identity between serotypes, where sequence differences can affect the capsid structures that are presented between serotypes and even subtypes (Acharya et al., 1989; Lea et al., 1994). VP1 nucleotide coding sequences are used for the genetic characterisation of FMDV strains due to their significance for virus attachment, protective immunity, and serotype specificity (Knowles et al., 2016).

Five antigenic sites have been identified for serotype O, including Site 1 which encloses the G-H loop and the C-terminus of the VP1 (Kitson et al., 1990; Crowther et al., 1993), serotype A (Saiz et al., 1991; Moore et al., 1989) and in SAT1 and SAT2 (Reeve et al., 2016; Grazioli et al., 2006). This site is considered an immunodominant antigenic site (Aggarwal and Barnett, 2002) and more than 25% of neutralising antibodies are thought to be directed towards this loop (Fernandez-Sainz et al., 2019). The G-H loop is located within B-strands G and H of the VP1 capsid protein (Logan et al., 1993) and encloses the receptor binding motif RGD (arginine-glycine-aspartic acid) which binds to the integrin receptor on host cells (Xie et al., 1987; Barnett et al., 1989). The G-H loop is a flexible structure, and molecular dynamic simulation demonstrates three states: (1) a relaxed reduced state on the virus capsid that is directed towards the three-fold axes, (2) a hanging protruding state as found in Fab-capsid complexes and (3) a structure directed towards the 2-fold axes. Both positions (2) and (3) occur when a disulphide bond is formed (Azuma and Yoneda, 2009). For example, the mobility of the G-H loop in serotype O is distorted from the hanged “up” position to a reduced “down” position due to a disulphide bond linking the cysteine 134 in the base of the loop to the cysteine 130 of VP2 (Logan et al., 1993).

Mabs D9 and B2 were raised against the O1 Switzerland 1965 (Lausanne) strain and bind to antigenic site 1 (Brocchi et al., 1983). These MAbs neutralise homologous FMDV isolates used in their production (McCullough et al., 1986). The D9 Mab has also been shown to bind to intact virions and synthetic peptides resembling the G-H loop for serotype O viruses (Xie et al., 1987), where five critical residues were identified; four in the G-H loop, leucine (L)-144, (L)-148 and lysine (K)-154, and one in the C-terminus at site 208 (Xie et al., 1987; Kitson et al., 1990). Shimmon et al., 2018 found a novel amino acid: aspartic acid (D)-147 (Shimmon et al., 2018). Substitution of the leucine at site 148 eliminated the ability of the D9 and B2 Mabs to neutralise (Shimmon et al., 2018). However, to our knowledge, there has not been any structured study including other serotypes to investigate the presence of cross-reactive epitopes within the G-H loop of FMDV.

## 2 Materials and methods

### 2.1 Peptide synthesis

Ten peptides were designed based on the full-length G-H loop: from residues 134–159 for O and A strains, 133–158 residues for SAT1 and 132–160 residues for SAT2. Eight peptides were synthesised for representative East African isolates from four serotypes: O/KEN/4/2018, O/ETH/9/2019, A/UGA/28/2019, A/SUD/9/2018, SAT1/TAN/22/2014, SAT1/TAN/22/2013, SAT2/ETH/16/2015, and SAT2/KEN/19/2017. In addition, two peptides one for O1Kaufbeuren (O1K) and one for A22/IRAQ/24/65 (A22) were synthesised; these peptides (31–35-mer) were derived from the G-H loop of FMDV (Table 1). Nine further overlapping 21-mer peptides (OP) were designed using the G-H loop sequence of O1K with a one amino acid overlap. Another series of 17-mer peptides derived from the O1K G-H loop of VP1 in which each residue was replaced by an alanine (A) were also produced and a 12-mer peptide lacking the last five amino acids (Table 2) (Burman et al., 2006). An additional three peptides, two for the G-H loop of SAT2/ZIM/7/83 with a variant containing ^145^RGDL^148^ and ^145^RGDM^148^ instead of ^145^RGDR^148^ (Table 3) and one for the G-H loop of A22 with serine instead of proline at site ^145^RGDXXP^150^, where also synthesised (Table 4). All peptides were synthesised by Peptide Protein Research Ltd, United Kingdom.

**Table 1:** Wild-type peptides used.

Parent virus	Amino acid sequence
O1Kaufberuen	^134^CRYNRNAVPNL**RGD**LQVLAQKVART^159^KKKKKK
A22 IRAQ 64/98	^134^TSKYSAGGTGR**RGD**LGPLAARVAAQ^159^KKKKKK
O KEN/4/2018	^134^CRYSSAPATNV**RGD**LQVLAQRVART^159^KKKKKK
O ETH/9/2019	^134^CKYGGVQATNV**RGD**LQVLAQKAART^159^KKKKKK
A UGA/28/2019	^134^TSRYSTATSGR**RGD**LGSLAARVATQ^159^KKKKKK
A SUD/9/2018	^134^TTKYTADTPPR**RGD**LGALAARLAAQ^159^KKKKKK
SAT1 TAN/22/2014	^133^YKPTSEAPRTNI**RGD**LATLAERIASE^158^KKKKKK
SAT1 TAN/22/2013	^133^YKPTSEAPRTNI**RGD**FAALAERIASE^158^KKKKKK
SAT2 ETH/16/2015	^132^NGECVYKKTPTAI**RGD**RAALAAKYAGSNH^160^KKKKKK
SAT2 KEN/19/2017	^132^NGECKYTDRVSAI**RGD**RTVLAAKYADSRH^160^KKKKKK

The bold values mean RGD sequence represents a conserved motif located both within the G-H loop and centrally in the peptide sequences.

**Table 2:** Alanine scanning O1K peptides.

Peptides	Amino acid sequence (17-mer)
WT	VPNL**RGD**LQVLAQKVAR
12-mer	VPNL**RGD**LQVLA-----
V1A	APNL**RGD**LQVLAQKVAR
P2A	VANL**RGD**LQVLAQKVAR
N3A	VPAL**RGD**LQVLAQKVAR
L4A	VPNA**RGD**LQVLAQKVAR
R5A	VPNLA**GD**LQVLAQKVAR
G6A	VPNL**R**A**D**LQVLAQKVAR
D7A	VPNL**RG**ALQVLAQKVAR
L8A	VPNL**RGD**AQVLAQKVAR
Q9A	VPNL**RGD**LAVLAQKVAR
V10A	VPNL**RGD**LQALAQKVAR
L11A	VPNL**RGD**LQVAAQKVAR
Q13A	VPNL**RGD**LQVLAAKVAR
K14A	VPNL**RGD**LQVLAQAVAR
V15A	VPNL**RGD**LQVLAQKAAR
R17A	VPNL**RGD**LQVLAQKVAA

The bold values mean RGD sequence represents a conserved motif located both within the G-H loop and centrally in the peptide sequences.

**Table 3:** SAT2 wild-type and mutated peptide.

Peptides	Amino acids sequences (17-mer)
RGDR	STAI**RGDR**AVLAAKYAN (wild-type)
RGDL	STAI**RGDL**AVLAAKYAN (mutated)
RGDM	STAI**RGDM**AVLAAKYAN (mutated)

The bold values mean RGD sequence represents a conserved motif located both within the G-H loop and centrally in the peptide sequences.

**Table 4:** A22 mutated peptide.

Peptides	Amino acids sequences (25-mer)
A22-S	TSKYSAGGTGR**RGD**LG**S**LAARVAAQKKKKKK (mutated)

The bold values mean RGD, sequence represents a conserved motif located both within the G-H loop and centrally in the peptide sequences. L, M, and R denote mutations.

### 2.2 Production of VP1 Mabs

Initial screening involved a panel of Mabs: for serotype O (D9 and B2) raised against strain O Switzerland 1965, for serotype A (3H2) against A5 Parma/ITL 1962, for SAT1 (HD7 and FC12) raised against SAT1 KEN 11/2005, and for SAT2 (3C5) raised against strain SAT 2 ZIM 5/81. These Mabs were obtained from Istituto Zooprofilattico Sperimentale della Lombardia e dell’Emilia Romagna (IZSLER) Brescia, Italy, and The Pirbright Institute, Surrey, United Kingdom. Mabs were derived from exhausted culture medium of hybridomas grown at high cells density and used in a dilution of 1/300 in all experiments.

### 2.3 In-house solid-phase enzyme linked immunosorbent assay

An indirect ELISA was developed using the G-H loop peptides. To determine the optimal coating concentration of the peptides, different concentrations were initially evaluated, ranging from 10 μg/mL to 1.25 μg/mL. The optimum coating concentration for the indirect ELISAs to give an appropriate dose response was found to be 5 μg/mL for the peptides used in the study (data not shown).

Briefly, plastic 96-well plates (Maxisorp; Nunc) were coated with 50 μL per well of the peptide in 0.05 M carbonate/bicarbonate coating buffer (pH 9.6) at 4°C overnight. Wells were then washed three times with phosphate-buffered saline (PBS) containing 0.1% Tween-20 (PBS-T) and patted dry. This washing step was repeated between all incubation steps. Wells were then blocked with 200 μL of blocking buffer [5% (wt/vol) skimmed milk–PBS-1% horse equine serum NZ 16050122 from Life Technologies Ltd.] at 37°C for 1 h. The plate was then incubated (37°C for 1 h) with 50 μL of Mab; the initial dilution was 1/300. The Mab was then 10-fold serial diluted in 5% blocking buffer down the plate. Then 50 μL of anti-mouse IgG HRP-conjugated secondary antibodies (A18751; Life Technologies LTD, United Kingdom) were diluted to 1/15,000 for in dilution buffer in 1% (wt/vol) skimmed milk–PBS. The chromogen development was mediated by the addition of 50 μL of HRP substrate (3,3′,5,5′-Tetramethylbenzidine; TMBW-0100-01, Sigma FAST; Sigma, United Kingdom). The reaction was stopped after 10 min. by the addition of 50 μL of 1 M sulphuric acid, and the optical density (OD) was measured at 450 nm wavelength using a SpectraMax^®^ ABS plate reader or a GloMax^®^ discover microplate reader. The relative binding percentage was calculated as the OD of the tested sample divided by the OD of the WT sample multiplied by 100.

### 2.4 Cell, virus propagation and virus neutralisation test

Virus neutralisation tests (VNTs) were carried out to test the ability of the D9 and B2 Mabs to neutralise different FMDV serotypes. IBRS-2 (pig kidney) cell line (Castro, 1964) from the World Reference Laboratory, The Pirbright Institute, was used for FMDV propagation and the virus neutralisation test. These cells were maintained in either Dulbecco’s modified Eagle’s medium or Dulbecco’s minimum essential medium (DMEM; Thermo-Fisher Scientific, United Kingdom) supplemented with 10% heat-inactivated adult bovine serum (BS; Thermo-Fisher Scientific, United Kingdom).

A 100-tissue culture infective doses 50% (100TCID_50_) of O/KEN/4/2018, O/ETH/9/2019, A/UGA/28/2019, A/SUD/9/2018, SAT1/TAN/22/2014, SAT1/TAN/22/2013, SAT2/ETH/16/2015, SAT2/KEN/19/2017, A/UGA/28/2019, O1K, and A22 were neutralised against D9 and B2 Mabs starting with the dilution of 1/600 in two-fold serial dilution. The results were reported as the final dilution required to neutralise 50% of the inoculated cultures (WOAH, 2022).

### 2.5 Statistical analysis for the assays

The comparison of the binding to different peptides in the ELISA was undertaken by applying an ANOVA with the Dunnett’s test in GraphPad Prism [version 9.4.1 (681)] to the binding percentages (OD of the tested sample divided by the OD of the WT sample multiplied by 100; Lee et al., 2022).

## 3 Results

### 3.1 D9 and B2 Mabs show serotype cross-reactivity

**Figure 1:**
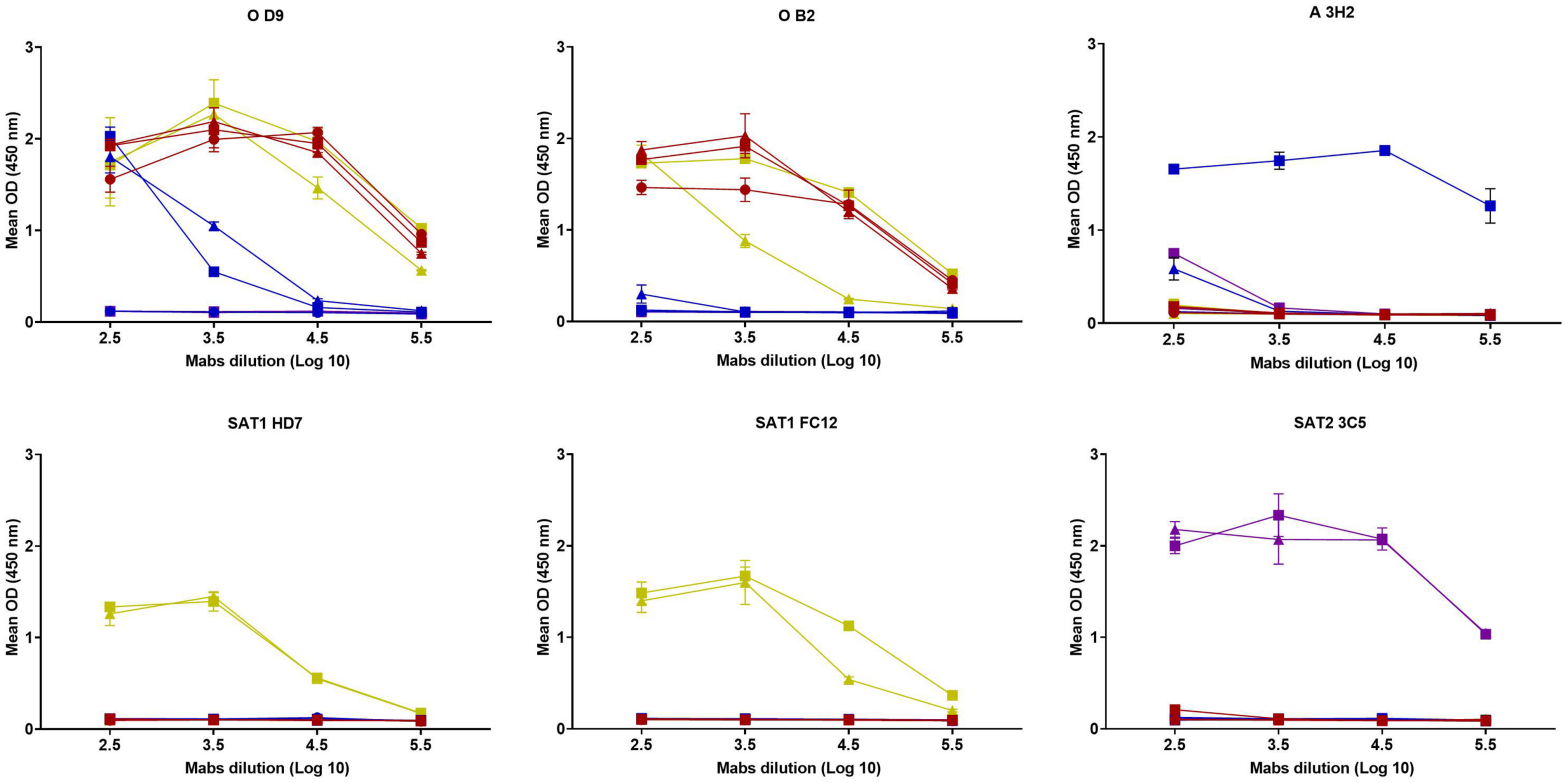
Reactivity of six Mabs (D9, B2, 3H2, HD7, FC12, 3C5) against G-H loop peptides from 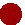 O1 KAUBEUREN, 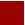 O/KEN/4/2018, 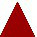 O/ETH/9/2019, 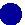 A22 IRQ/24/64, 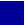 A/UGA/28/2019, 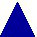 A/SUD/9/2018, 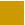 SAT1/TAN/22/2014, 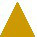 SAT1/TAN/22/2013, 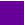 SAT2/ETH/16/2015 and 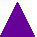 SAT2/KEN/19/2017. The error bars represent the range of duplicate determinations.

D9 Mab reacted to three serotype O peptides, two serotype A peptides from East African viruses with a curve approximately 100-fold weaker, two serotype SAT1 peptides but not the two serotype SAT2 peptides or a peptide designed for A22 Iraq (Figure 1). In contrast, the B2 Mab reacted only to the three serotype O peptides and two peptides from serotype SAT1, one of them showed approximately 10-fold lower reactivity, whilst other Mabs including those for serotype A (3H2), SAT1 (HD7 and FC12) and SAT2 (3C5) were serotype specific.

### 3.2 Neutralisation activity of D9 and B2 Mabs

VNTs were performed to determine the neutralisation activity of the D9 and B2 Mabs. The neutralisation titres for D9 using O1K and O KEN/4/2018 were 3.7log_10_; O ETH/9/2019 was 2.8log_10_ and 3.4log_10_ for SAT1 TAN/22/2014 and below the limit of detection (0.9log_10_) for the remaining FMDV isolates: A22, A/UGA/28/2019, A/SUD/9/2018, SAT1/TAN/22/2013, SAT2/ETH/16/2015 and SAT2/KEN/19/2017 whilst B2 Mab only neutralised O1K and O KEN/4/2018 with a titre greater than 2.4log_10_.

### 3.3 Characterising the binding site of the D9 and B2 Mabs using G-H loop overlapping peptides

**Figure 2:**
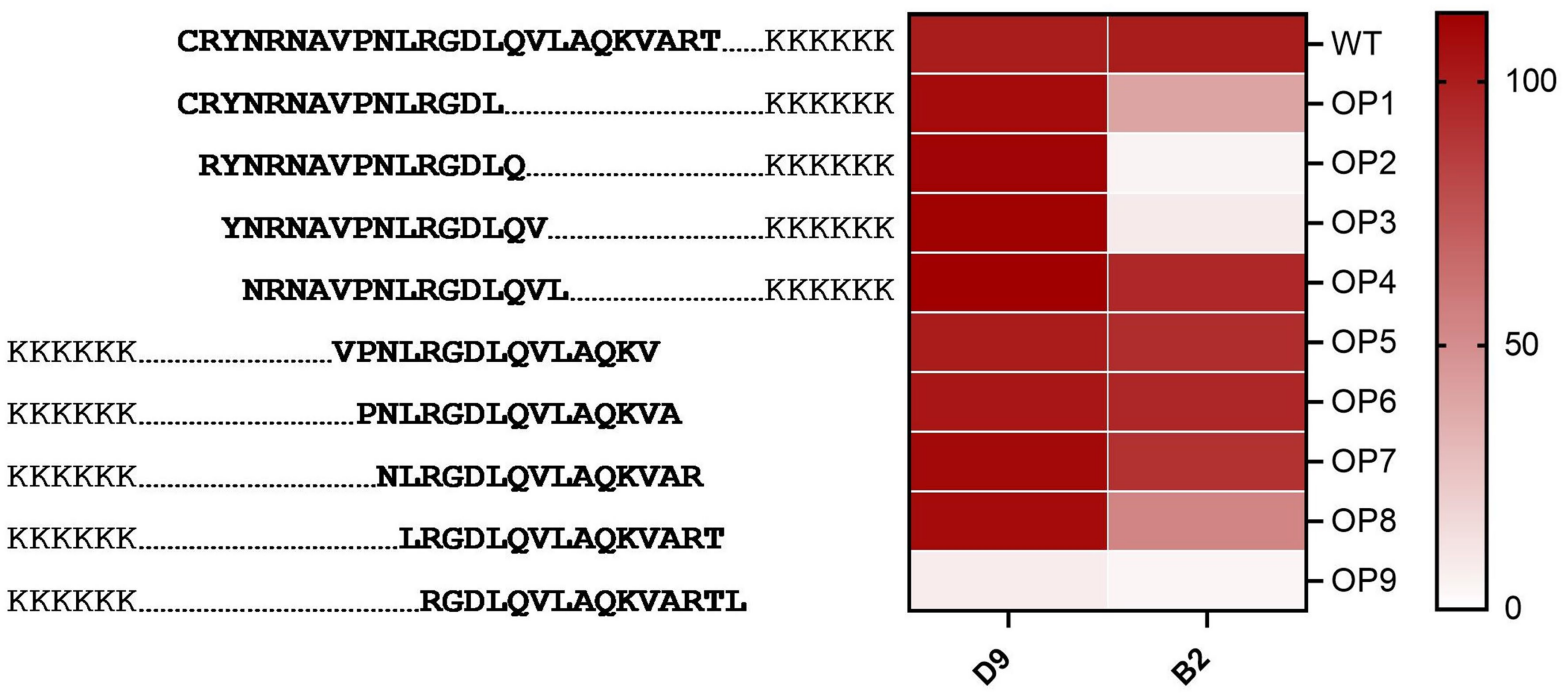
Characterising the binding region of D9 and B2 Mabs using O1K overlapping peptides. Red colour gradient ranges from pale to deep red according to the intensity of binding.

To define the region that is detected by the D9 and B2 Mabs, a set of overlapping G-H loop peptides from O1K was used. These peptides were designed with one amino acid increments starting from the N terminus. D9 Mab bound with an increase in relative binding percentage for OP3 and OP4 than the WT and a significant reduction (*p* < 0.0001) for OP9 (Figure 2). Whilst B2 had a significant reduction (*p* < 0.0001) than the wild-type when bound to OP2, OP3 and OP9 by 95, 91 and 96%, respectively, it decreased by 60 and 46% for OP1 and OP8, respectively, and a reduction of 9% for OP7 (see Figure 2).

### 3.4 Characterisation of the critical amino acid residues using alanine scanning O1K peptides

**Figure 3:**
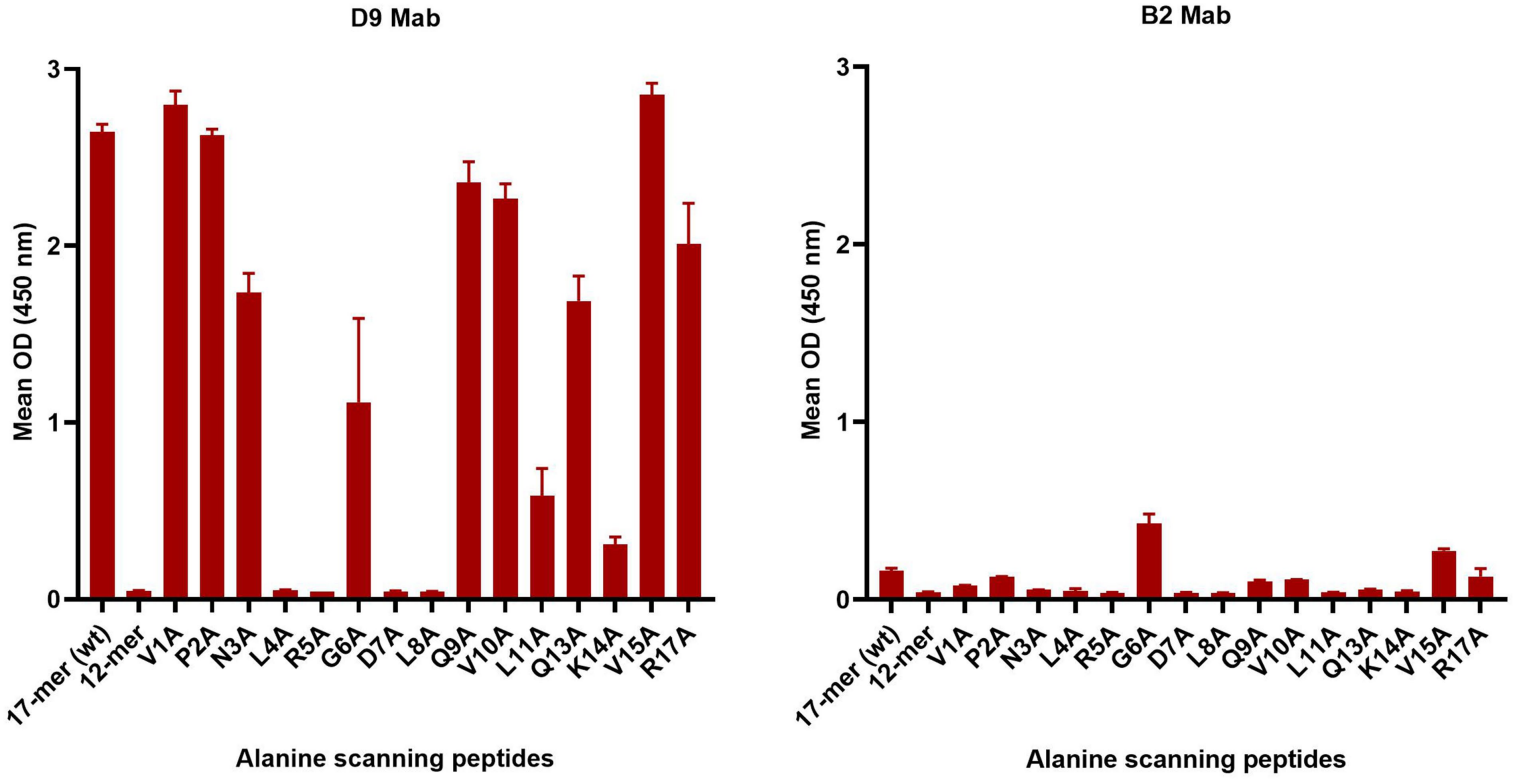
Mab D9 and B2 reactivity profiles against the alanine scanning O1K peptides compared to the WT (17-mer) and truncated WT (12-mer) that lacks five residues at the C-terminus. The error bars represent the range of duplicate determinations.

To further investigate the critical amino acids important for D9 and B2 Mab binding, a set of peptides with one alanine replacing the existing amino acid sequentially from the N terminus was tested (Table 2 and Figure 3). D9 Mab bound to all these peptides, except L4A, R5A, G6A, D7A and L8A with a significant decrease (*p* < 0.0001) in the relative binding compared to the WT (17-mer) by 34, 36, and 24% for N3A, Q13A, and R17A, respectively and by 60, 78 and 88% for G6A, L11A and K14A, respectively and no binding against the 12-mer peptide. In contrast, B2 was poorly bound to all of these peptides, where the strongest signal was achieved for G6A and V15A, which was much lower than the D9 responses.

### 3.5 Determination of the effect of a single amino acid substitution on Mab binding and epitope structure

**Figure 4:**
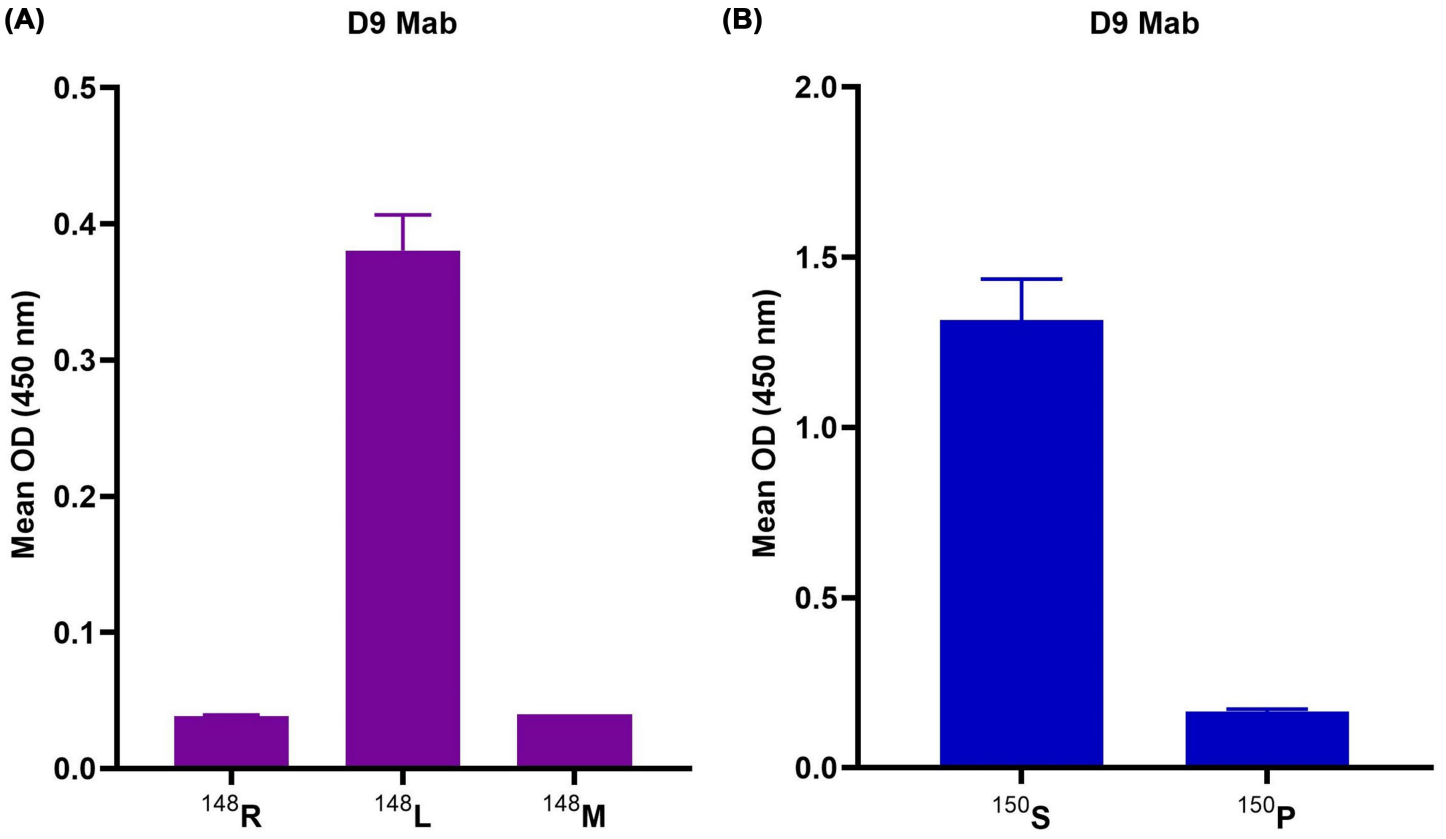
**(A)** D9 Mab reactivity against three SAT2 peptides: ^145^RGDL^148^, ^145^RGDR^148^ and ^145^RGDM^148^ (purple) and **(B)** D9 reactivity against A22 (blue) ^145^RGDLGP^150^ and mutated peptide ^145^RGDLGS^150^. The error bars represent the range of duplicate determinations.

To determine the effect of the type of an amino acid for D9 and B2 Mabs. D9 Mab was tested against two modified SAT2 peptides each with a single amino acid substitution from the WT ^145^RGDR^148^ to ^145^RGDL^148^ (the most abundant amino acid in this site) or ^145^RGDM^148^ (the second most abundant amino acid; see Figure 4A). D9 Mab shows a significant increase for ^145^RGDL^148^ binding when compared to the wild-type ^145^RGDR^148^. As expected, B2 poorly binds to these peptides with similar reactivity across the three peptides (data not shown). Furthermore, the D9 and B2 Mabs were tested against two mutant A22 peptides with a single amino substitution the ^149^GP^150 149^GS^150^. D9 binding was increased for the ^150^S peptides compared to the ^150^P peptide (Figure 4B). These peptides did not bind to B2 (data not shown).

## 4 Discussion

This study used a panel of VP1-specific Mabs that bind the G-H loop of FMDV to identify serotype cross-reactive epitopes. Two Mabs (D9 and B2) that bound G-H loop peptides for more than one serotype were selected for further study. The D9 Mab reacted against peptides derived from three serotype O isolates (O1K, O/KEN/4/2018 and O/ETH/9/2019), two serotype A isolates (A/UGA/28/2019 and A/SUD/9/2018) and two SAT1 isolates (SAT1/TAN/22/2014 and SAT1/TAN/22/2013), but not two SAT2 specific peptides (for isolates SAT2/ETH/16/2015 and SAT2/KEN/19/2017). D9 binding to the two A East African isolates was 100-fold less reactive and could be a result of the presence of arginine instead of leucine at site 144. D9 binding for these different serotype peptides was evident even though these peptides had amino acid substitutions at sites that have been previously identified as critical for D9 reactivity such as site 144 (from leucine to valine in O strains and to isoleucine in SAT1), site 148 (from leucine to phenylalanine) and site 154 (from lysine to arginine). B2 bound only to serotype O and SAT1 peptides and had a different binding profile against SAT 1 peptides with 10-fold weaker binding for SAT1/TAN/22/2013, which could be due to the presence of phenylalanine instead of leucine at position 148.

Li et al. (2024) identified the conserved “RGDL” motif on VP1 as a primary target of porcine cross-serotype broadly neutralising antibodies (bnAbs), suggesting it may mimic integrin receptor recognition and represent a cross-serotype neutralising antigenic site in FMDV. Whilst RGDL appears to elicit cross-neutralising responses in pigs, this is not always the case, as observed using the murine-derived Mabs. Notably, although the RGDL motif is present in the VP1 peptide sequences of serotype A isolates A/UGA/28/2019 and A/SUD/9/2018, the monoclonal antibody D9 failed to fully neutralise A strains [below the limit of detection (0.9log_10_)]. This highlights potential differences in immunological recognition between porcine and murine systems and highlights that binding and neutralisation requirements could vary depending on the conformation of the neutralising epitope.

Serotype-specific neutralising epitopes have been identified within the G-H loop of VP1 for serotype SAT2. However, the precise amino acid residues critical for this epitope remain undefined. In this study, the SAT2-specific neutralising MAb 3C5, described by Grazioli et al. (2006), bound exclusively to SAT2-derived peptides, suggesting the presence of a neutralising epitope unique to each serotype within the G-H loop.

These data demonstrate the inter-serotypic cross-reactivity of D9 and B2. A previous study has shown that D9 could opsonise serotype A viruses (Summerfield et al., 2022). Additional Mabs (*n* = 4) in the screening panel had serotype-specific responses that were restricted SAT1, SAT2 and A peptides.

The D9 and B2 Mabs were further tested using targeted overlapping serotype O G-H loop peptides, alanine scanning peptides and mutant peptides. Four previously described amino acids were found to be essential for D9 Mab binding including 144L, 147D, 148L, and 154K within the G-H loop (Kitson et al., 1990; Xie et al., 1987; Shimmon et al., 2018). Data from the overlapping peptides confirmed the importance of these sites whilst the binding site for B2 Mab was found to be located in the region upstream of the RGD since there was a decrease in binding when the first three amino acids were removed from the peptides; This finding was supported by the data from the alanine scanning peptides where poor binding was observed when the first three amino acids were lacking. A better binding of B2 to the peptide with alanine replacing the asparagine at site 146 (^145^RAD^147^) was found when compared to the B2 Mab binding behaviour for other peptides. The alanine scanning peptides were used to further identify the critical amino acids for D9 where a new critical amino acid was identified in close proximity to the RGD, position 145 R.

Despite the presence of amino acids previously considered critical for D9 binding (144 L, 145 R, 147 D, 148 L and 154 K), D9 did not bind to the WT A22 peptide. This suggests that the presence of a proline at the RGD + 3 site, which is known as a helix breaker in the A22 peptide sequence, alters the epitope integrity and the presentation of G-H loop epitopes (Ludi et al., 2014). The impacts of proline residues on antigenicity have been previously observed for other serotype A viruses (Ludi et al., 2014). This hypothesis was tested by using a peptide that had a modification at this site (proline to serine) where D9 binding to the modified peptide (S) was enhanced compared to to the A22 WT peptide (P). In addition, B2 does not appear to bind to serotype A, which again suggests that binding is affected not only by the absence of critical amino acids but also by the epitope structure and integrity changes.

SAT2 WT peptides failed to bind to D9. However, D9 binding was observed when the SAT2 peptide sequence was engineered to contain a substitution from arginine to leucine (R148L) the most highly represented residue at the critical amino acid RGD + 1 site for peptides tested in this study. Methionine and leucine are non-polar, hydrophobic amino acids; however, methionine is a sulphur-containing amino acid, which provides extra flexibility that may affect the binding of D9 (Aledo, 2019). Furthermore, the presence of arginine, a positively charged, hydrophilic residue, could introduce electrostatic interactions or conformational changes that may further influence the structural integrity or binding affinity of D9 (Armstrong et al., 2016).

The lower OD signal detected in these assays is likely due to the lower solubility of these peptides. McCullough et al. (1986) reported that D9 Mab does not bind to the G-H loop sequence of A_24_ Cruzeiro, findings which are perhaps explained by the presence of methionine at RGD + 1 (^134^TSKYAVGGSGRRGDMGSLAARVVKQ^156^).

These results characterise a cross-reactive and neutralising D9-like epitope on the G-H loop of FMDV which is also present in intact virions for some, but not all, FMDV serotypes as demonstrated by the neutralisation responses of this Mab.

This study focuses on the impact of defined substitutions at critical sites. However, it should be noted that there is variability in the amino acid sequences at these positions for the wild-type G-H loop sequences. This suggests that the D9 and B2 epitopes can accommodate further amino acid substitutions that were not tested in this study. This work provides insights into the nature of cross-reactive epitopes located on or close to the G-H loop of VP1. These findings help understand why serological assays used to measure FMDV-specific antibodies have poor serotype specificity (Ludi et al., 2022). In conclusion, our results indicate the binding ability of Mabs to a representative G-H loop peptides derived from various FMDV serotypes. Further testing of individual mutations using a reverse genetic system could be conducted to confirm the effects observed for the mutant peptides in this study and fully appreciate all of the component epitopes that contribute to this cross-reactivity, which may lead to the rational design of peptides with improved serotype specificity.

## Data Availability

The original contributions presented in the study are included in the article/supplementary material, further inquiries can be directed to the corresponding author.
